# An ecological role for assortative mating under infection?

**DOI:** 10.1007/s10592-017-0951-9

**Published:** 2017-03-27

**Authors:** L. J. Campbell, M. L. Head, L. Wilfert, A. G. F. Griffiths

**Affiliations:** 1 Environment and Sustainability Institute, University of Exeter, Penryn Campus, Penryn, Cornwall TR10 9FE UK; 2 Institute of Zoology, Zoological Society of London, Regent’s Park, London, NW1 4RY UK; 3 Division of Evolution, Ecology and Genetics, Research School of Biology, Australian National University, Canberra, ACT Australia; 4 Centre for Ecology and Conservation, University of Exeter, Penryn Campus, Penryn, Cornwall TR10 9FE UK; 5 FoAM Kernow, Studio E, Jubilee Warehouse, Commercial Road, Penryn, Cornwall TR10 8FG UK

**Keywords:** Disease, Assortative mating, Genetic diversity, Conservation threat, Mate choice, Reproductive fitness, Immunity, Pathogens, Parasites

## Abstract

Wildlife diseases are emerging at a higher rate than ever before meaning that understanding their potential impacts is essential, especially for those species and populations that may already be of conservation concern. The link between population genetic structure and the resistance of populations to disease is well understood: high genetic diversity allows populations to better cope with environmental changes, including the outbreak of novel diseases. Perhaps following this common wisdom, numerous empirical and theoretical studies have investigated the link between disease and disassortative mating patterns, which can increase genetic diversity. Few however have looked at the possible link between disease and the establishment of assortative mating patterns. Given that assortative mating can reduce genetic variation within a population thus reducing the adaptive potential and long-term viability of populations, we suggest that this link deserves greater attention, particularly in those species already threatened by a lack of genetic diversity. Here, we summarise the potential broad scale genetic implications of assortative mating patterns and outline how infection by pathogens or parasites might bring them about. We include a review of the empirical literature pertaining to disease-induced assortative mating. We also suggest future directions and methodological improvements that could advance our understanding of how the link between disease and mating patterns influences genetic variation and long-term population viability.

## Introduction

One of the core tenets of conservation genetics is the need to maintain and manage genetic diversity at both local population and species wide levels. Often species of greatest conservation concern exist in small, isolated and fragmented populations which are directly associated with a loss of genetic diversity (Furlan et al. 2012). In turn this is known to reduce the evolutionary adaptive potential of a population, rendering it less capable of an evolutionary response to fluctuations in its environment (Willi et al. 2006). In a vicious cycle, those species which may be experiencing, or have experienced, a reduction in population size are those which are most endangered by further potential environmental stochasticity (McCallum and Dobson 1995). One such environmental fluctuation is the introduction of a new disease into an ecosystem.

Currently zoonotic infectious diseases are emerging at an ever-increasing rate, and are more numerous than at any known prior point in history (Jones et al. 2008). Diseases have been implicated in the global declines of numerous species, including those of great conservation concern, for example various diseases in Florida panther (Roelke et al. 1993), Chytridiomycosis and Ranavirosis in amphibians (Daszak et al. 1999; Green et al. 2002), various infective agents in Californian sea lions (Acevedo-Whitehouse et al. 2003), White-Nose syndrome in bats (Blehert et al. 2009), Devil facial tumour disease in Tasmanian Devils (Lachish et al. 2011). Although current empirical evidence of disease alone causing local or global extinction is very limited (Smith et al. 2006), it is theoretically acknowledged as a possibility and several potential risk factors have been identified. These include: (i) a frequency dependent or vector borne transmission route (see Box 1); (ii) small or fragmented host populations; and (iii) the presence of reservoir host species (De Castro and Bolker 2005). There are many examples of disease acting as a contributing cause of species or population extinction (Smith et al. 2009) and it is considered highly likely that the role of disease in extinctions is under appreciated in the current literature (Pedersen et al. 2007).

**Box 1:** Ordinary (OTD) and sexually (STD) transmitted diseases

It is not always clear whether a pathogen is an OTD or a STD and there are several examples where the same pathogen can be transmitted by either route (Smith and Dobson 1992). The route that serves a pathogen best is determined by the structure of the population that it is infecting. OTD transmission is in most cases limited by the density of a population. Transmission via air, general contact or faecal-oral ingestion etc. is more likely to occur between individuals existing at higher densities. STDs and those transmitted via a vector are more likely to have a frequency dependent transmission route. This is not limited by the density of the population, but by the frequency of infected individuals within the population and the success of those individuals to obtain matings (in the case of STDs). As such, STDs are able to persist within a population at lower densities (Lockhart et al. 1996). It should be noted that the traditional density vs frequency dependent transmission dichotomy assumes that the contact networks of a population is homogeneous, i.e. each individual is as likely to contact any other individual. In reality this is rarely the case and this can lead to a less obvious optimum transmission route (Bansal et al. 2007). It is currently theorised that frequency dependent diseases pose the greatest conservation threat. Ordinary, density dependent diseases that cause high mortality will reduce the population density to such a level that their transmission is hindered and the pathogen itself will become locally extinct before the host population (Smith et al. 2009). Frequency dependent transmitted diseases do not reach such a threshold and therefore have the potential to drive a species extinct. The selection pressures on hosts for characteristics such as high genetic variation, effective immunity or avoidance will be similar for both OTD and STD (Lockhart et al. 1996). However, because frequency dependent diseases like STDs require their host to successfully infect other individuals, there is a greater pressure on them to remain undetectable. For example STDs that are overly virulent and cause obvious loss of condition or competiveness in their host are less likely to be transmitted (Knell and Webberley 2004). It is therefore less likely that frequency dependent diseases will lead to the establishment of non-random mating patterns, as they are often less virulent and possess a longer latent stage during which time they can be transmitted to but not easily detected by other reproductive members of a population. STDs are touted as a possible cause for the evolution of monogamy (Aral and Leichliter 2010; Bauch and McElreath 2016), however mathematical models have shown that the relationship between STDs and mating systems is more complex and that the optimal mating strategy depends on many factors (Thrall et al. 1997). STDs may also influence mating systems by altering host behaviour towards promiscuity (Lockhart et al. 1996) or increasing the attractiveness of infected individuals (Thrall et al. 1997)	

The relationship between population genetic structure and population fitness, including susceptibility to disease, is well established (Sherman et al. 1988; Burger and Lynch 1995; Lacy 1997; Altizer et al. 2003) and one of the fundamental determinants of a populations genetic diversity and structure is the mating pattern that it exhibits (Wright 1950). Numerous studies have investigated disassortative mating patterns that potentially increase the individual and population genetic diversity at immune related loci and how this increase in genetic diversity can potentially mitigate the impacts of disease. However, recent evidence suggests that an assortative mating strategy may be advantageous when a host is faced with a particularly virulent pathogen (Teacher et al. 2009a) or when homozygosity leads to greater resistance (Nuismer et al. 2008). Indeed mathematical models suggest that the conditions under which disassortative mating can evolve appear to be more stringent than previously thought—occurring only when the costs associated with discrimination between potential mates are low and heterozygosity infers greater resistance to an infecting pathogen (Nuismer et al. 2008).

A comprehensive search of the current literature (ISI Web of Science, keywords: assortative mating, mating pattern, disease, infection) revealed that to date, relatively few empirical studies have investigated the potentially important role that pathogens or parasites can have in establishing assortative mating patterns (Table 1). In this paper we outline the interaction between infection, mating patterns and population genetics, as well as summarise how non-random mating patterns may form in response to disease - including a full review of empirical evidence for disease-induced assortative mating, and highlight the potentially important broad scale genetic implications of assortative mating patterns. We ask whether methodological oversights in the scant previous research mean that the prevalence of infection-induced assortative mating patterns has previously been underestimated, and suggest ways that this could be addressed in future work.

**Table 1:** A summary of the empirical case studies of disease induced assortative mating presented here. Papers gathered from ISI Web of Science using the keywords: assortative mating, mating pattern, infection, disease

Pathogen species	Host species	Laboratory	Field	Paper
*Gyrodactylus turnbulli*	*Poecilia reticulata*	***✓***		Houde & Torio (1992)
*Pomphorhyncus laevis*	*Gammarus pulex*	***✓***		Poulton & Thompson (1987)
*Pomphorhyncus laevis* + *P.minutus*	*G.pulex*	***✓***	***✓***	Bollache et al. (2001)
*Polymorphus paradoxeus* + *P.marillis*	*Gammarus lacustris*	***✓***	***✓***	Zohar and Holmes (1998)
*Various (mainly Haemoproteus lanii)*	*Lanius collurio*		***✓***	Votypka et al. (2003)
*Trypanosoma sp*	*Ficedula hypoleuca*		***✓***	Ratti et al. (1993)
*Microphallus papillorobustus*	*Gammarus insensibilis*		***✓***	Thomas et al. (1995)
*Coccidiasina sp*	*Meleagris gallopavo*	***✓***		Buchholz (2004)
*G.turnbulli*	*P.reticulata*	***✓***		Lopez (1999)
*Crytocotyle sp*	*Syngnathus typhle*	***✓***		Mazzi (2004)
*Ormia ochracea*	*Gryllus lineaticeps*	***✓***		Beckers and Wagner (2013)

## Interactions between genetic diversity and disease

Genetic diversity is the term used to describe the variety in genetic composition at a given ecological level, and can be discussed in terms of communities, species, populations and individuals. Across all levels the literature generally indicates that the higher the genetic diversity present, the better the chances of survival in the face of an environmental change. For example, individuals with high genetic variation are heterozygous at a large number of genetic loci. Increased levels of within individual genetic variation are desirable as low heterozygosity has long been associated with a number of conditions that negatively impact both fitness and survival (Charlesworth and Charlesworth 1987). The increased fitness of heterozygotes is most often attributed to the masking of deleterious recessive alleles by the presence of another dominant allele, or the innate benefit derived from being heterozygous at specific loci, often termed heterozygote advantage or overdominance (Crow 1948).

Similarly, a population with more genetically dissimilar individuals may experience lower vulnerability to extinction due to stochastic events or environmental change (Crow 1948; Burger and Lynch 1995; Lacy 1997). For example, a population with high genetic variation at immune genes has a higher chance of containing individuals that are resistant to novel infections, potentially mitigating the impact of an emergent pathogen or parasite (Woelfing et al. 2009). Further, diseases have been shown to spread more quickly (Altermatt and Ebert 2008) and infect more readily (Ganz and Ebert 2010) in less genetically variable populations due to a higher percentage of the population being susceptible to infection.

The elevated fitness of more genetically diverse populations when faced with infection was first noticed in agricultural crops. The so called “monoculture effect”, where the intensive management of crops to improve yield results in decreased genetic diversity, is well known to heighten the susceptibility of plant populations to diseases (Zhu et al. 2000; Mundt 2002). The need to maintain between individual genetic variation in order to protect population genetic diversity is thought to be a reason behind the evolution of polyandry and polygyny in insect colonies (Sherman et al. 1988; Liersch and Schmid-Hempel 1998) and is also sometimes cited as a possible reason for the maintenance of sexual reproduction itself (Hamilton et al. 1990). The relationship between genetic diversity, disease susceptibility and disease spread has recently been thoroughly reviewed by King and Lively (2012) .

We have seen how the genetic variability of an individual or population can influence a host’s ability to cope with a disease outbreak. However, disease can also influence the genetic variability of its host in a number of ways. Population bottlenecks caused by a disease outbreak can result in substantially lowered host genetic diversity. Even in the absence of high mortality the genetic constitution of subsequent generations of populations can be influenced by infection. For example, reproductive fitness can be harmed both directly by the infective agent and/or by the costs imposed by the host’s own immune defences. Disease may impact upon an individual’s ability to produce offspring, e.g. by sterilisation (Givens and Marley 2008) and increased investment in immunity can result in reduced investment in other fitness related traits (Lochmiller and Deerenberg 2000). Changes such as a reduction in brood size (Poulton and Thompson 1987) and decreased offspring survival (Bonneaud et al. 2003) may also occur in individuals that carry a pathogen burden.

## Mating patterns, genetic structure and disease

One less considered way that disease can influence the genetic diversity of its host is through causing direct or indirect changes to the host’s mating system. The pattern in which matings are distributed in a population is a key-contributing factor in determining the stability of a population’s genetic structure. The mating patterns of sexually reproducing organisms can be grouped into one of three broad categories: random, assortative and disassortative mating (Wright 1921). Box 2 outlines more information on these patterns and on the basic principles of the relationship between mating pattern and population genetic structure.

**Box 2:** How mating p atterns influence genetic diversity

One measure of a population’s genetic variation is the number of heterozygotes that it contains. The number of heterozygotes in a population is not static and is dependent on a number of factors, one of which is the mating pattern. The following is a simplified overview of how mating patterns can affect the genetic diversity of a population. It should be noted that mating patterns are themselves subject to selection and form only a part of the potentially complex mating system of a population, which may also depend on criteria such as sex ratios, relative reproductive output and differential parental investment between the sexes (See; Emlen and Oring 1977; Shuster 2009 for more detail). Whole mating system evolution occurs over a far extended time scale to the potential population genetic changes seen in response to disease **Random** mating assumes no mating preference at all—this is a core assumption of Hardy–Weinberg Equilibrium. Over time the numbers of heterozygotes within a population remain roughly stable **Assortative** mating patterns occur when individuals that have a particular allele at a given locus will be more likely to mate with other individuals that also possess that allele. There is an elevated chance that offspring will be homozygous for that allele. The number of heterozygotes within a population decreases over time **Disassortative** mating patterns are the exact opposite of assortative mating in that an individual that does not possess a particular allele is more inclined to mate with an individual that does. There is a greater chance that progeny will be heterozygous at that locus, and between generations genetic diversity increases	

Disease can cause the establishment of non-random mating patterns in a number of ways. Firstly, non-random mating patterns can be maintained via an active choice bias from one or both sexes in favour of either more similar or more different partners. This relies on the ability to read phenotypic clues of a potential partner’s genotype, that is to say, that there is a detectable cue upon which to base mate choice. Such mate choice behaviour in response to disease is a key mechanism of sexual selection (Hamilton and Zuk 1982). Discrimination against diseased or low quality mates can result in direct benefits to the choosy sex, including increased availability of resources (via pairing with a mate better able to compete for them), higher levels of parental care (Reynolds and Gross 1990), and the direct evasion of infection by avoiding association with infected individuals (Borgia 1986). The Hamilton Zuk hypothesis (1982) suggests that showy secondary sexual characteristics demonstrate an absence of significant parasitic burden and that choosy individuals may be seeking out mates with advantageous, possibly resistant, genes to confer a fitness advantage on their offspring. Mate choice may lead to both assortative and disassortative mating patterns (see sections below).

Even in the absence of an overt mate choice bias an individual’s chance of obtaining matings can be hampered by phenotypic changes resulting from infection such as a reduction in body size (Olsson 1993) or degradation of secondary sexual ornamentation (Houde and Torio 1992) that may make individuals less competitive. The erosion of quality in such fitness related traits can also result in the formation of non-random mating patterns simply because those individuals susceptible to disease are less able to secure matings than those free from infection.

In the following section we will consider empirical examples of how disease induces shifts in mating patterns away from random mating. Since review papers on disease and disassortative mating are relatively plentiful we briefly summarise the links between disease and disassortative mating, while focusing our attention on a more thorough review of the empirical literature pertaining to disease induced assortative mating. Perhaps due to differing selection pressures (see Box 1), empirical evidence from sexually transmitted infections (STIs) resulting in the formation of assortative mating patterns is lacking. As such, the case studies presented below do not include any examples where the infective agent is exclusively sexually transmitted.

## Disease induced disassortative mating

Much of the literature that examines the links between disease and mating patterns focuses on disassortative mating. Disassortative mating is thought to represent an adaptive response to the presence of diseases because it increases the genetic diversity within offspring, thus there is an increased likelihood that at least some members of the next generation will possess a genotype that confers resistance to established or novel pathogens. Infecting organisms are less likely to be adapted to successfully infect individuals that harbour rare alleles, so exposure to infections may result in a selection pressure on the host that favours rare alleles (Borghans et al. 2004). Similarly, mating for diversity when under attack by a pathogen or parasite may provide additional advantages to hosts by providing a non-static genome for pathogens to evolve against (Potts et al. 1994), helping to keep the host one step ahead of its pathogen or parasite in the evolutionary “arms race”, an example of the Red Queen Hypothesis (Van Valen 1973).

It is well known that immune genotype and associated chemo-olfactory cues can influence mate choice and therefore major histocompatibility complex (MHC) mediated disassortative mating has been reviewed in detail more than once (e.g. Jordan and Bruford 1998; Penn and Potts 1999). The MHC (a collection of genes that are integral to the vertebrate immune system’s ability to deal with pathogens, Trowsdale 1993) has been linked to disassortative mate choice in many different species (Jordan and Bruford 1998; Freeman-Gallant et al. 2003; Huchard et al. 2010; Eizaguirre et al. 2011). An individual’s MHC genotype is often advertised by pheromones (Wedekind et al. 1995) but it has also been shown that humans may be able to infer MHC compatibility in a partner by appearance (Roberts et al. 2005). Mating for diversity at the MHC may have evolved as a mechanism to reduce inbreeding in populations and to boost the genetic diversity of progeny (Potts and Wakeland 1993). Many organisms select mates based on a pheromone profile that is different from that associated with their immediate relatives or other individuals encountered early in their lives (Penn and Potts 1999).

## Disease-induced assortative mating

Despite the apparent prevalence of disassortative mating patterns based upon MHC genotype, it has been suggested that when a population is subject to a particularly virulent or persistent pathogen, assortative mating for a favourable genotype may be a more effective response.

Assortative mating based upon the MHC is proposed as a potential explanation for observed changes in the population genetics of European common frog (*Rana temporaria*, IUCN least concern). Microsatellite genotyping of wild populations that were either under infection or free from a lethal viral pathogen (*Ranavirus*) revealed that infected populations undergo a loss of genetic variation but do not show signs of inbreeding. This is indicative of an assortative mating pattern (Teacher et al. 2009a). The same populations also show signs of directional selection at the MHC (Teacher et al. 2009b) suggesting that the MHC could act as a basis for assortative mate choice. Globally, *Ranavirus* has been implicated in the declines of many species of amphibians, fish and reptiles (Cunningham et al. 1996; Whittington et al. 2010; Marschang 2011), including some of conservation concern, like the dusky gopher frog (Sutton et al. 2014). They are considered such a threat to wildlife that the World Organisation for Animal Health has classified the type species, Frog virus 3, as a notifiable disease. If the changes in mating systems observed in the common frog are seen in other species, their threat to population viability may be greater than currently understood. Reduced genetic diversity within populations brought about by assortative mating patterns could make populations more vulnerable to future outbreaks of *Ranavirus* and other diseases or stochastic events.

Visual cues such as colour intensity are often used to infer a potential mate’s disease status. Behavioural studies conducted on Poeciliid fish offer good examples of how choosy individuals discriminate against infected potential partners. In the Trinidadian guppy (*Poecilia reticulata*, not Red List assessed), mate choice can be influenced by the strength of carotenoid pigmentation in male fish. Males that had been exposed to the parasite *Gyrodactylus turnbulli* had significantly reduced orange colour intensity and appeared duller. Females that were given a choice between uninfected and infected males in a laboratory trial were found to preferentially mate with uninfected males (Houde and Torio 1992). Colour has also been shown to be an important indicator of pathogen burden and a trait for mate choice selection in birds (Sundberg 1995).

Such discrimination against infected individuals may at first glance seem to benefit a population that is experiencing infection. Avoiding contact with infected individuals will lower pathogen transmission and the number of individuals that are susceptible to infection in subsequent generations is likely to reduce due to the increased mating success of individuals resistant to infection. However, with the exception of some sex specific infections, it is likely that both sexes within a population will be infected simultaneously. Assortative mating patterns based upon susceptibility to infection can therefore form due to a loss of preference for healthy mates by the choosy sex when they themselves are infected (Poulin and Vickery 1996). In such situations, diseased or susceptible individuals are not prevented from mating entirely but instead will mate with other susceptible individuals, creating pairs based upon susceptibility to infection. In instances where infection rates are high among a population and the heritability of resistance to the infecting agent is high, the potential for subsequent change in the population genetic structure of a population is high. Empirical evidence for such scenarios again comes from behaviour observed in female Trinidadian guppies (*P.reticulata*) infected with *G.turnbulli*. In laboratory mate choice trials both infected and naïve focal females were given a choice between an infected and a naïve male. Infected females spent significantly more time soliciting mating attempts from the infected males, which suggests a loss of choosiness due to their own infection status (López 1999). Although *P.reticulata* is unevaluated by the IUCN, a number of its congeners, including the sulphur molly (*P.sulphuraria*) and the broad spotted molly (*P.latipunctata*) are listed as critically endangered. The evaluation of potential mating pattern changes in these species could form a useful part of their conservation strategy. A similar loss of choice in infected individuals has also been observed during empirical studies on wild turkeys (*Meleagris gallopavo*, IUCN least concern) infected with a coccidian species (Buchholz 2004) and male pipefish (*Syngnathus typhle*, IUCN least concern) infected with a *Cryptocotyle* trematode (Mazzi 2004).

Pairs based on the resistance or susceptibility to an infecting pathogen or parasite can be formed in a number of additional ways. In the absence of explicit choice for healthy mates, assortative mating can occur by a direct loss of reproductive fitness in both sexes of a population concurrently. Infection by blood parasites of the genus *Haemoproteus* resulted in a reduction in fitness of red backed shrike (*Lanius collurio*, IUCN least concern). Infected female shrikes arrived later to the wild breeding sites than uninfected females. Infected males displayed larger melanin spots on their tails, which act as an honest signal of parasite load and male quality, allowing females to choose mates of the highest possible quality. The authors found an assortative pattern of pair formation in regards to infection status. This was suggested to be the result of the infected females arriving late to the breeding sites when only the low quality infected males were still unpaired (Votýpka et al. 2003).

Delayed time of breeding was also observed in the pied flycatcher (*Ficedula hypoleuca*, IUCN least concern but decreasing population trends) during field studies. Males infected with *Trypanosoma* species arrived later to the breeding sites than uninfected males and were found to be in poorer general condition as judged by morphometric measures of quality such as wing feather length. It is again suggested that the late arriving, low quality males will compete for low quality females as those of higher quality will have already formed pairs (Rätti et al. 1993), again resulting in the possible formation of pairs assortative for susceptibility to disease.

Pathogens and parasites can also in some cases directly manipulate the behaviour of their host. Such behavioural manipulation can result in infected individuals no longer being able to mate with uninfected individuals within a population. This is especially true of parasites, which will often modify the behaviour of their intermediate host to make them more vulnerable to predation by their ultimate host, facilitating transfer. A good example of this is seen in the amphipod *Gammarus insensibillis* (not IUCN assessed) that is often subject to heavy parasitism from the trematode *Microphallus papillorobustus*. Thomas et al. (1995) demonstrated that a strong bias for infected individuals to pair together exists in parasitised populations. This is suggested to be the result of a direct behavioural change brought about by the parasite which forces infected individuals to reside much higher in the water column, where predation is much more likely. Uninfected individuals remain at lower depths so opportunities for individuals of differing infection status to pair are reduced.

Under such assortative mating patterns, variation in heritable resistance to infection will be lost due to a rise in the number of individuals with homozygous genotypes for either resistance or susceptibility. If the infection is lethal then susceptible individuals could be lost entirely from the population. The decrease in susceptible individuals will negatively impact the spreading potential of the parasite or pathogen infecting the population thus increasing host viability in the short term (King and Lively 2012). However, the reduction of diversity in immune genotype will render the population more susceptible to novel diseases in the future (O’Brien and Evermann 1988; Spielman et al. 2004). The reduced diversity will also lower the adaptive potential of a population in response to other environmental changes (Burger and Lynch 1995; Lacy 1997).

As such, disease-induced assortative mating should be of particular interest to those studying the conservation of endangered species in the face of environmental change.

## Gaps in the existing research

As discussed above, the long-term implications for populations affected by assortative mating could be profoundly negative and as such we believe this to be an important area of study that warrants further and renewed empirical research effort. In this section we review gaps in existing research literature, some of which could mean that the frequency of occurrence of disease-induced assortative mating and the threat posed to vulnerable species could currently be underestimated.

### A need to investigate the impact of disease on both sexes

There are species in the literature that demonstrate how the apparent impact of an infection upon a mating system can vary based upon which sex is the focal animal of a study. Female Trinidadian guppies were explicitly shown by Houde and Torio (1992) to preferentially solicit matings from unparasitised healthy males. However it was then shown by Lopez (1999) that females who were themselves parasitised lacked the choosiness of their uninfected counterparts. While the findings of Houde and Torio suggest directional selection under infection in favour of healthy males, when these two studies are considered together it is clear that infection could result in the formation of potentially damaging assortative mating patterns, i.e. healthy females still discriminate in favour of healthy males, and unhealthy individuals mate with each other. This could result in population genetic changes similar to those seen by Teacher et al. (2009a) in common frogs.

A further example is seen in literature pertaining to the shrimp *Gammarus pulex*. Poulton and Thompson (1987) showed that male mate choice was significantly influenced by the parasitic load of females. It was later observed by Bollache et al. (2001) that the parasitic load of males themselves dictated their likelihood of obtaining preferred matings. Again, the Poulton and Thompson findings suggest selection against infected females, however when taken together the two studies are suggestive of assortative mating brought about by a loss of fitness in both sexes when under infection.

These two examples make it clear that in order to fully understand how a pathogen affects the mating ecology of a population it is imperative to study the impact that it has on both sexes. At present, few species have received investigation into how infection influences the mating behaviours of both sexes and therefore there is potential that assortative mating patterns may be more prevalent than the current literature suggests.

### A lack of non-laboratory study systems

In their review of MHC disassortative mating, Penn and Potts (1999) suggest that the inferences that can be drawn from mating behaviours in laboratory experiments are limited. The manipulation of an organism’ surroundings and environment take any observed behaviours out of context and as such extrapolation of results into wild populations could be considered spurious. This viewpoint is supported by Fisher et al (2015), who following their comparative field and laboratory experiments into boldness behaviours in wild crickets found that behavioural measurements were not replicable between the two theatres of observation.

Further issue has been found with laboratory-based mating experiments in that they often utilise a dichotomous design, where focal animals are given a direct choice between just two stimulus animals (Wagner 1998). In many species such a stark choice of mates is rarely the case (Buchholz 2004; Nelson et al. 2013). There is little cost to a choosy individual when the choice is between two often widely disparate individuals in a uniform setting such as a mate choice tank. In the wild, choices are often based on several criteria and will often be in competition with other “choosers”, making choosiness far more costly (Alatalo et al. 1988). It has also been found that observed preference of a choosy sex for one focal animal over another does not translate into differences in the reproductive fitness of the two stimulus animals (Zala et al. 2015).

Despite these shortcomings the majority of studies in the field of infection induced assortative mating rely only on data collected in the laboratory (Table 1). Reasons for conducting laboratory work are many; observation of mating behaviours is often easier under laboratory conditions and it is also often less labour intensive and cheaper than fieldwork that seeks to answer similar questions. Laboratory studies also allow for hypotheses to be tested directly by the control and manipulation of experimental variables. However, it is clear that an over reliance of laboratory studies when looking into the impact of pathogens on mating systems may lead to its importance in the wild being misunderstood. In addition, species of conservation concern are inherently less likely to be studied due to restrictions on collection for laboratory experiments. In light of this, future effort should seek to utilise or incorporate field-based systems wherever possible.

The current field based literature comes from either avian or gammarid systems. Birds often pair up and remain on nests for an extensive breeding season, display parental care and can easily be identified using ringing techniques, meaning that reproductive fitness can be measured directly by brood size/fledgling success. Gammarid shrimp species, though aquatic, occur in shallow water and display prolonged breeding behaviours such as mate guarding, which are easily observed in the field to infer mate preference. The number of eggs laid can again be used as a measure of reproductive fitness. As such, these systems lend themselves more easily to field based research than do others. Advances in technology that are being used in other areas of behavioural research offer opportunities for improving experimental designs to allow them to more closely resemble wild conditions. For example, breeding populations of many species could be more accurately recorded by using technology (Sumner et al. 2007)/software (Green et al. 2012) capable of tracking the movement and association patterns of multiple animals independently over an extended period, allowing the inclusion of several focal and stimulus animals in each trial.

In the wild, populations may be subject to infection by multiple different agents at any one time (Petney and Andrews 1998). Laboratory research allows for the exclusion of infective agents that are not of interest. If studying the impact of particular pathogen on a population makes the use of wild study systems intangible, then the use of more elaborately designed enclosures or mesocosms that more realistically mimic the habitat of the focal species should be considered. A good example of such a system has been utilised in the study of infection and mating patterns in mice in very large enriched enclosures (Nelson et al. 2013; Zala et al. 2015). Another option would be to increase the use of field systems that are monitored over long periods of time for presence/absence of disease. The use of such systems allow for the selection of study populations of known infection status, meaning that infected vs. uninfected “treatment” groups can be incorporated into the study design. The UK based system for the study of amphibian disease is a good example of such a system (see Teacher et al. 2009a, b; North et al. 2015; Price et al. 2015) and similar efforts are also being made in response to Devil facial tumour disease in Tasmania (Coupland and Anthony 2007). Although the formation of such study systems in remote or less well populated areas would require greater expenditure in terms of research effort and finance, the degree to which they allow for comparative studies between populations of differing, known disease histories to be performed should warrant them due consideration.

### The co-evolutionary perspective

The co-evolution of hosts and parasites plays a large role in how a population or species copes with an infection. For example, populations may be less resistant to non-locally occurring isolates of the same pathogen that normally infects them (Ridenhour and Storfer 2008). This suggests that a co-evolutionary history allows host and pathogen to accumulate adaptions that better able them to counter the defences of the other, leading to an eventual equilibrium. Little attention however has been given expressly to the co-evolutionary interactions between host mating pattern and the pathogens with which they can be infected. According to Borgia’s parasite avoidance hypothesis (Borgia 1986), changes to a mating system, particularly those based on a choice biased against infected individuals, will result in a decreased spread of pathogens that are especially virulent and incapacitate or maim their host to a such a degree that they are easily selected against. This applies a selection pressure on the pathogen towards lower virulence, so as not to impact the reproductive potential of the host too greatly. Supporting evidence for this comes from recent modelling work that suggests for the first time that all interactions between pathogens and host mating systems should be viewed as entirely co-evolutionary (Ashby and Boots 2015). This is a sea change in our understanding of how diseases impact their host mating systems and presently co-evolutionary interactions are not widely incorporated into epidemiological models of disease spread.

To date, there is a lack of empirical appraisal of this theory. Addressing this is potentially critical for fully understanding the conservation implications of disease induced assortative mating. Future work should make a concerted effort to evaluate the true impact of disease induced shift in population mating ecology by combining the disciplines of evolutionary biology, behavioural ecology and epidemiology - not only studying how infection can affect host mating biology, but also how the disease-induced changes to mating biology can impact on the spread of the causative pathogen. The inclusion of additional variables into experimental designs such as both a local and non-local pathogen strain, or host and pathogen samples taken at different time-points over a long-term study, would allow for the exploration of co-evolutionary dynamics in the establishment of assortative mating patterns. Experimental exploration of the effect of assortative mating patterns on the spread of disease would require more complex methodological approaches but could potentially be achieved using model systems with short generation times, existing within mesocosms. Such systems have been used to study the impact of genetic diversity on disease transmission in insects (Liersch and Schmid-Hempel 1998; Hughes and Boomsma 2004). The expansion of these systems to incorporate vertebrate species would require significant experimental effort but is by no means impossible and would undoubtedly prove extremely useful in advancing knowledge in this study area.

### A need to investigate species of conservation concern

All of the focal species of empirical case studies presented in this review are listed by the IUCN as “Least Concern” or are unevaluated. Given the growing number of cases where threatened species are subjected to infectious diseases (Lyles and Dobson 1993; Roelke et al. 1993; Acevedo-Whitehouse et al. 2003; Spielman et al. 2004) understanding the impact of infection mediated assortative mating patterns on the genetics of vulnerable species is paramount. Knowledge of how the mating patterns of threatened species are impacted by infectious disease should be considered important information for conservation when evaluating all of the potential threats that a species faces. The experimental exploration of such patterns in endangered species warrants special importance but must also be treated sensitively. As we have stated above, much of the empirical work in this area is laboratory based. In the absence of established captive breeding programmes this requires the removal of animals from the wild for use in controlled experiments. This is obviously undesirable in species where wild population numbers are already under threat. The establishment of controlled, wild field systems as mentioned above is one possible solution to this problem. As is the extrapolation of findings from a non-threatened closely related species to the threatened species of interested, provided their ecology is known to be broadly similar. The deliberate infection of endangered species for laboratory studies also raises additional ethical concerns meaning that field based study systems not requiring experimental infections should be utilised exclusively if possible.

## Conclusions

Assortative mating, be it by active mate choice or passive changes to competitiveness, can benefit a population by allowing faster adaptation against pathogens and parasites that are particularly virulent and persistent within an environment. It can however result in a loss of genetic diversity from the population at large. Reduced genetic diversity is well known to increase the vulnerability of populations to environmental stochasticity (Willi et al. 2006). Species that are of great conservation concern have often been shown to be at increased risk of disease due to their already depleted genetic diversity (Roelke et al. 1993; Siddle et al. 2007; McCallum 2008; Lachish et al. 2011). The potential for the establishment of assortative mating patterns means that diseases may reduce the genetic diversity within an infected population, even when mortality or impacts on individual fecundity are low. It is therefore possible that through the establishment of assortative mating patterns, the conservation threat posed by infectious disease may be greater than currently acknowledged. While the implications of disease-induced assortative mating are significant, there is a dearth of appropriate empirical research into the phenomenon, which may be more common than currently realised (Nuismer et al. 2008). A number of factors including some of human influence mean that there are more new diseases emerging now than ever before (Jones et al. 2008). The increasingly regular emergence of new, highly virulent diseases (Cunningham et al. 1996; Daszak et al. 1999; McCallum 2008; Blehert et al. 2009) means that it is vital that we apply modern techniques and renewed interdisciplinary research effort to understanding how these new diseases may affect host-breeding biology. Empirical evaluation of disease-induced assortative mating is particularly pressing in those species of greatest conservation concern which are subjected to infectious diseases and may require further or altered management as a result.
